# A novel posterior multiple screws distraction reducer system versus anterior release, posterior internal distraction, and subsequent spinal fusion for severe scoliosis

**DOI:** 10.1186/s12891-021-03963-w

**Published:** 2021-02-05

**Authors:** Ganjun Feng, Yong Huang, Leizhen Huang, Yongliang Wang, Juehan Wang, Chunguang Zhou, Lei Wang, Zhongjie Zhou, Xi Yang, Limin Liu, Yueming Song

**Affiliations:** Department of Orthopedic Surgery and Orthopedic Research Institute, West China Hospital, Sichuan University, Chengdu, 610041 Sichuan China

**Keywords:** Severe scoliosis, Surgical treatment, Multiple screws distraction reducer system, Internal distraction, Comparison

## Abstract

**Purpose:**

We previously reported anterior release, posterior internal distraction, and subsequent spinal fusion (ARPIDF) for the correction of severe scoliosis with a satisfactory correction rate. However, surgical procedures were completed in 2–3 stages. Here we compare Cobb angle of ≥90° in scoliosis correction between a novel posterior multiple screws distraction reducer (MSDR) system and ARPIDF.

**Methods:**

Thirty-six patients with severe scoliosis treated by MSDR or ARPIDF (*n* = 18 in both groups). We retrospectively analyzed and compared outcome measures between the two groups over a minimum follow-up duration of 2 years. The following variables were compared between the two groups: age at surgery, sex, etiology, flexibility of the main thoracic curve, number of fused segments and screws, operation time, estimated blood loss, hospitalization time, follow-up duration, various radiological parameters, complication rate, and Scoliosis Research Society-30 score.

**Results:**

There were no significant between-group differences with respect to age, sex, etiology, flexibility of the main thoracic curve, number of fused segments and screws, and follow-up duration. Further, there was no significant difference in terms of preoperative, postoperative, and final follow-up findings of the radiographic data. However, the ARPIDF group had longer operation and hospitalization times and greater blood loss. In the ARPIDF group, 4 patient developed complications (infection, intraoperative neuromonitoring changes, transient dyspnea); none of these events occurred in the MSDR group.

**Conclusion:**

The use of MSDR helped achieve greater scoliosis correction with a shorter operation time, lower blood loss, and lower complication rate than the use of ARPIDF. MSDR facilitates safer and easier correction of severe scoliosis without increasing surgical risk.

## Introduction

The surgical treatment of severe scoliosis is technically challenging and potentially risky [1–3]. Various correction methods, such as posterior instrumentation combined with anterior release [4–6], combined anterior and posterior surgical procedures with halo traction [7, 8], vertebral column decancellation [9], and combined anterior and posterior instrumentation [10], have been reported to date. In recent years, an increasing number of surgeons have advocated posterior-only vertebral column resection (PVCR) for the treatment of severe scoliosis [11–13]. However, aggressive osteotomies with PVCR entail a greater risk of neurologic injury and intraoperative blood loss.

To achieve safe scoliosis correction, we performed staged anterior release, posterior internal distraction, and subsequent spinal fusion (ARPIDF) for the treatment of severe scoliosis [4, 6, 14–16]. A satisfactory scoliosis correction rate was achieved with this method. However, two- or three-stage surgery increases treatment cost and risks associated with anesthesia and prolonged hospitalization time, which also increase the potential infection rate. We developed a novel device to achieve scoliosis correction in a safe and more effective manner. In this approach, provisional rods are placed on the concave side of the spine proximally and distally; these rods are then linked to an external distraction reduction device termed multiple screws distraction reducer system (MSDR). The purpose of the current study was to introduce this novel device and compare MSDR with ARPIDF for severe scoliosis (Cobb angle ≥90°) with respect to radiographic outcomes, operation time, intraoperative blood loss, complication rate, and Scoliosis Research Society (SRS)-30 scores.

## Methods

### Inclusion criteria and grouping

Data pertaining to patients with severe scoliosis who were surgically treated at a single institution were retrospectively reviewed after obtaining institutional review board approval. All patients provided written informed consent, and the study protocol was approved by the Ethics Committee of West China Hospital of Sichuan University. All methods and procedures in this study were performed in accordance with the relevant guidelines and regulations of ethics committee of Sichuan University. The main thoracic curve of all patients had a Cobb angle of ≥90°, and flexibility was < 30% using bending films. Patients were excluded from analysis if the postoperative follow-up duration was < 2 years.

A total of 36 patients were divided into two groups based on the two correction techniques. In the MSDR group (*n* = 18), scoliosis was corrected using MSDR. In the ARPIDF group (*n* = 18), scoliosis was corrected using anterior release with temporary posterior internal distraction followed by posterior spinal fusion and instrumentation.

### Radiographic measurements and clinical results

Data pertaining to the following variables were compared between the two groups: age at surgery, sex, etiology, flexibility of the main thoracic curve, number of screws, operation time, estimated blood loss, hospitalization time, follow-up duration, various radiological parameters, and complication rate.

All radiographs were evaluated digitally using the synapse analysis system. Measurements were determined using preoperative and postoperative follow-up radiographs. The flexibility of the main thoracic curve was calculated according to Cobb angle obtained using bending films. Radiographic analysis included Cobb angle measurement of the main thoracic curve, thoracic kyphosis, coronal balance, and sagittal balance. Thoracic kyphosis was measured using the Cobb method from the superior end plate of T5 to the lower end plate of T12. Coronal balance was measured as the distance between the C7 plumb line and central sacral vertical line. Sagittal balance was measured as the distance between the C7 plumb line and posterosuperior corner of S1. Shoulder imbalance were measured by shoulder height difference which defined as the height difference of the soft tissue shadows directly superior to the acromioclavicular joints. Radiological parameters were measured at the following time points: preoperatively, immediate postoperatively, and at the most recent follow-up.

All patients preoperatively and postoperatively completed SRS-30. Mean scores for domains including pain, function, self-image, mental health, and satisfaction as well as total score were calculated preoperatively and at postoperative follow-up.

Data were analyzed using IBM SPSS Statistics (IBM Corp., 2013, Armonk, NY, USA). Continuous variables are presented as mean and standard deviation. Two-tailed independent *t*-test, Wilcoxon rank sum test, and analysis of covariance analysis were used to compare the two groups. A *p*-value of < 0.05 was considered indicative of statistical significance.

### MSDR correction technique

**Fig. 1:**
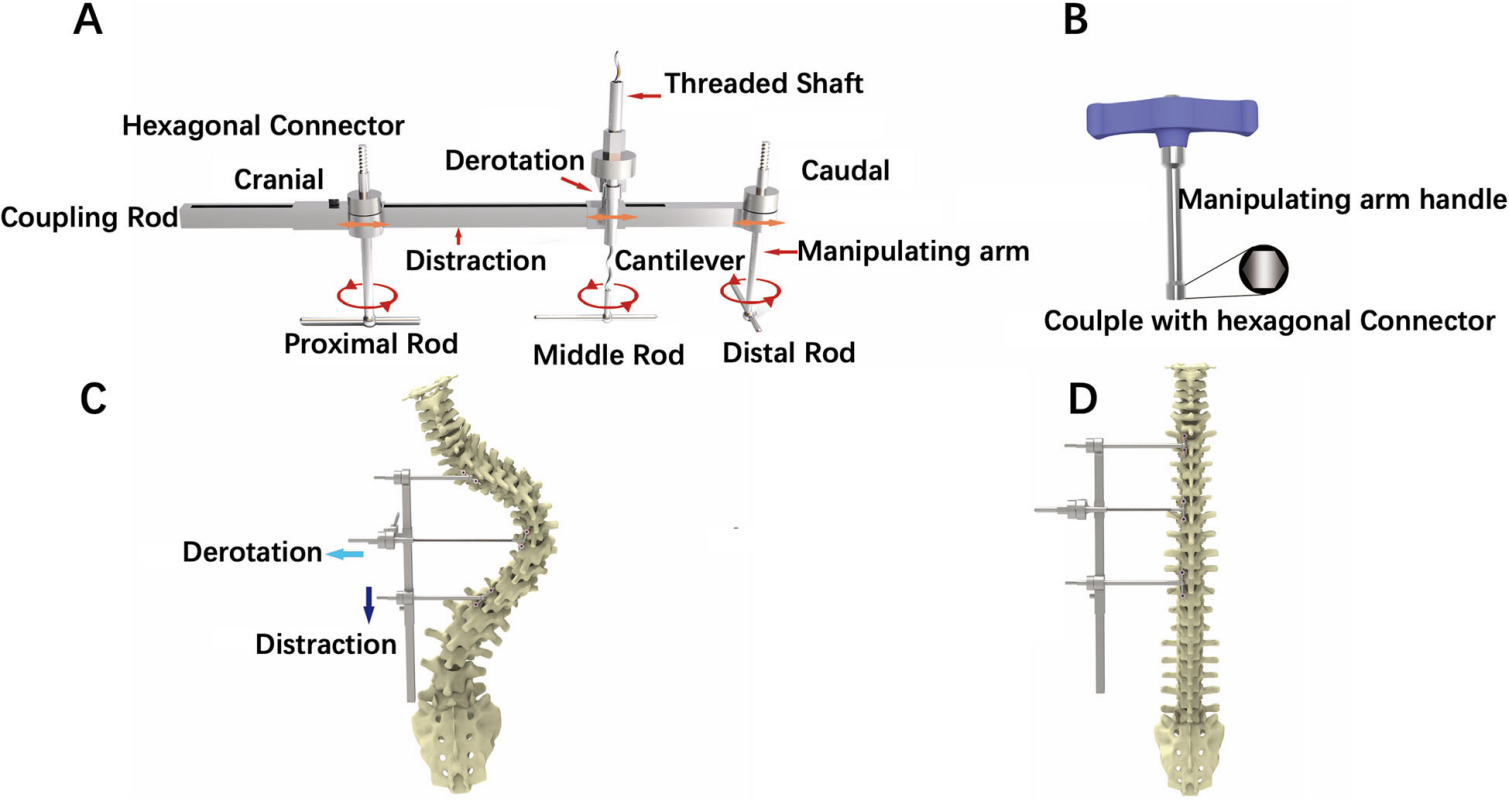
Posterior rod-link-distraction-reducer (MSDR) system. **a** A coupling rod connected to distraction and derotation couplers; Three provisional rods are placed proximal, apical, and distal to the apex. Two manipulating arms and a cantilever are attached to the provisional rods and then linked to the coupling rod; these are adjusted using the distraction and derotation couplers. The manipulating arms and cantilever are used by the surgeon to correct deformities in the three planes. The significant lever arm forces generated allow the surgeon to easily and simultaneously derotate, translate, and correct the spine in the coronal, sagittal and axial planes. **b** Manipulating handle couples with the hexagonal connector to lock the manipulating arms. **c** MSDR provides correction force for distraction, derotation, and restoration of thoracic kyphosis. By using multiple screws connected with proximal rods, the correction forces are averagely distributed to 6–8 screws. The risk of screws loosening or failure during deformity correction process is significantly decreased. **d** Once correction has been achieved, the construct is rigidly locked and a permanent rod is placed and secured on the contralateral side

**Fig. 2:**
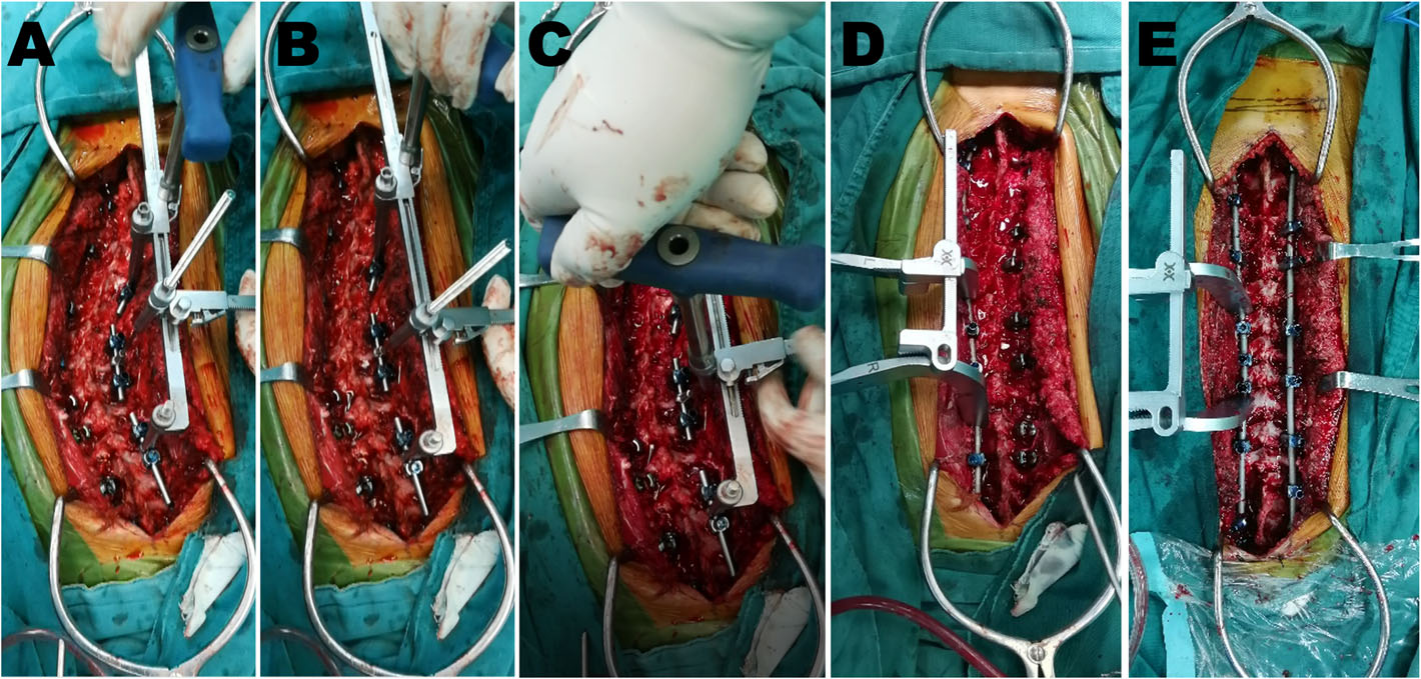
Procedures of MSDR correction technique: **a** With the spine exposed posteriorly, pedicle screws are inserted. Provisional rods are attached to the proximal, apical, and distal segments on the concave side of the spine. **b** The first maneuver addresses the coronal deformity with consistent distraction between the proximal and distal segments. Multiple rounds of distraction can be performed to take advantage of the viscoelastic properties of biological tissues. **c** A derotation coupler enables axial derotation of the spine and rib cage. The shaft connected with a cantilever lifts the middle provisional rod to restore thoracic kyphosis. The second step is to restore thoracic kyphosis and is performed by rotating the derotation coupler through a threaded shaft. The shaft connected with a cantilever lifts the middle provisional rod. Axial derotation of the spine and rib cage is also achieved in this step. **d** Once sufficient correction has been achieved, the permanent rod is measured, cut, and undercontoured for the convex side of the spine to push down toward the apex and improve rotational deformity. Spinal compression on the apical segments is also performed for further coronal correction. **e** The permanent rod is measured, cut, and contoured for the concave side of the spine. Overcontouring the concave rod in thoracic kyphosis helps further restore the thoracic sagittal plane. The apical segments can be translated posteriorly to correct thoracic hypokyphosis and laterally to correct coronal deformity

MSDR is shown in Fig. 1. With the spine exposed posteriorly, pedicle screws are inserted. Multiple Ponte osteotomies around the apical vertebrae were performed. Provisional rods are attached to the proximal, apical, and distal segments on the concave side of the spine. Then, the provisional rods may be contoured according to the lumbar lordosis at the distal end and the upper thoracic deformity at the proximal end. After the provisional rods are secured by tightening set screws, the manipulating arms of MSDR are inserted over the provisional rods (Fig. 2a). A threaded shaft is also connected to the apical provisional rod. Next, one coupling rod with sawtooth is placed onto the manipulating arms to connect the proximal and distal manipulating arms. The first maneuver addresses coronal deformity with consistent distraction between the proximal and distal segments. Multiple rounds of distraction can be performed to take advantage of the viscoelastic properties of biological tissues (Fig. 2b). The second maneuver aims to restore thoracic kyphosis and is performed by rotating a hexagonal connector through the threaded shaft. The shaft connected with a cantilever lifts the middle provisional rod (Fig. 2c). Axial derotation of the spine and rib cage is also achieved in this step. Once sufficient scoliosis correction is achieved, the permanent rod is measured, cut, and undercontoured for the convex side of the spine to push down toward the apex and improve rotational deformity. Spinal compression on the apical segments is also performed for further coronal correction (Fig. 2d).

Once the convex permanent rod is captured with locking caps, MSDR and provisional rods are removed. The permanent rod is measured, cut, and contoured for the concave side of the spine. Overcontouring the concave rod in thoracic kyphosis helps further restore the thoracic sagittal plane (Fig. 2e). The apical segments can be translated posteriorly to correct thoracic hypokyphosis and laterally to correct coronal deformity. Any compression, distraction, or in situ bending may be performed prior to the final tightening of the construct.

### ARPIDF correction technique

ARPIDF was performed as previously reported [15, 17]. Briefly, anterior release included an approach involving the convex side of the area to be resected through a thoracic incision. Intervertebral discs were fully excised, and vertebral mobility was verified by rotating a Cobb elevator in the disc space. After anterior release, the patient was placed in the prone position for internal distraction. Two pedicle screws were inserted at the cephalad level and two at the caudad level of the major coronal curve on the concave side. Two rods were placed at the cephalad and caudad fixation points separately and connected with a side-by-side connector. Subsequently, internal distraction was performed under spinal cord monitoring. Posterior spinal fusion with multiple Ponte osteotomies around the apical vertebrae was performed 1–4 weeks after the initial surgery. The previously implanted rods were replaced with contoured permanent rods on the convex and concave sides of the spine. 6.0 mm titanium alloy rod was used in both techniques.

## Results

The MSDR group included 10 females and 8 males. Their mean age at surgery was 18.4 ± 2.8 years. There were 15 cases of idiopathic scoliosis (5 cases of type Lenke 2, 3 cases of type Lenke 3 and 7 cases of type Lenke 4) and 3 of neuromuscular scoliosis. The mean flexibility of the main thoracic curve was 14.8% ± 4.2%. The ARPIDF group included 11 females and 7 males. Their mean age at surgery was 19.2 ± 2.5 years. There were 16 cases of idiopathic scoliosis (6 cases of type Lenke 2, 2 cases of type Lenke 3 and 8 cases of type Lenke 4) and 2 of neuromuscular scoliosis. The mean flexibility of the main thoracic curve was 12.1% ± 3.8%. There were no significant between-group differences with respect to age, sex, etiology, Lenke curve classification of idiopathic scoliosis and flexibility of the main thoracic curve (Table 1).

**Table 1:** Comparison of patient characteristics between the two groups

	MSDR (*n* = 18)	ARPIDF (*n* = 18)	*p*-value
Age (years)	18.4 ± 2.8	19.2 ± 2.5	0.41
Sex			0.97
Male	8	7	
Female	10	11	
Etiology			0.98
Idiopathic	15	16	
Lenke curve classification (I\II\III\IV\V\VI)	(0\5\3\7\0\0)	(0\6\2\8\0\0)	0.85
Syringomyelia associated	3	2	
Flexibility of the main thoracic curve (%)	14.8 ± 4.2	12.1 ± 3.8	0.96
Number of fused segments	13.4 ± 1.3	13.7 ± 1.2	0.48
Number of screws	18.8 ± 2.2	17.9 ± 3.1	0.32
Operation time (min)	287.5 ± 24.5	563.0 ± 34.2	0
Estimated blood loss (mL)	1024.8 ± 148.2	1320.5 ± 237.4	0.007
Hospitalization time (days)	7.9 ± 2.1	14.9 ± 3.0	0
Follow-up duration (years)	2.8 ± 0.4	2.9 ± 0.5	0.82

*MSDR* multiple screws distraction reducer, *ARPIDF* anterior release, posterior internal distraction, and subsequent spinal fusion

The mean number of fused segments in the MSDR and ARPIDF groups was 13.4 ± 1.3 and 13.7 ± 1.2, respectively; the mean number of screws used in the MSDR and ARPIDF groups was 18.8 ± 2.2 and 17.9 ± 3.1, respectively; the between-group difference in this two respects was not statistically significant (Table 1). In the MSDR group, the mean operation and hospitalization times were 287.5 ± 24.5 min and 7.9 ± 2.1 days, respectively; the mean estimated blood loss was 1024.8 ± 148.2 mL. In the ARPIDF group, the mean operation and hospitalization times were 563.5 ± 34.2 min and 14.9 ± 3.0 days, respectively; the mean estimated blood loss was 1320.5 ± 237.4 mL. The ARPIDF group had longer operation and hospitalization times and greater estimated blood loss (Table 1). The mean follow-up duration in the MSDR and ARPIDF groups was 2.8 ± 0.4 and 2.9 ± 0.5 years, respectively; the between-group difference in this respect was not statistically significant.

In the MSDR group, the preoperative main thoracic curve of 96.2° ± 5.9° was corrected to 22.9° ± 3.5° at the immediate postoperative curve assessment; this corresponded to 76.5% ± 6.8% scoliosis correction (Table 2). At the most recent follow-up, the main thoracic curve was 25.1° ± 3.7°, which corresponded to 74.1% ± 8.8% scoliosis correction. There was a 2.5% loss of correction compared with the immediate postoperative curve measurement. In the ARPIDF group, the preoperative main thoracic curve of 97.3° ± 6.5° was corrected to 21.1° ± 2.4° at the immediate postoperative curve assessment; this corresponded to 78.0% ± 9.2% scoliosis correction. At the most recent follow-up, the main thoracic curve was 24.6° ± 4.1°, which corresponded to 75.1% ± 10.5% scoliosis correction. There was a 2.8% loss of correction compared with the immediate postoperative curve measurement. However, there was no significant between-group difference with respect to the preoperative main thoracic curve and loss of correction. In addition, the two groups showed similar main thoracic curve and correction rate postoperatively and at the most recent follow-up.

**Table 2:** Comparison of radiographic data between the two groups

	MSDR	ARPIDF	*p*-value
Cobb angle of the major curve (°)			
Preoperative	96.2 ± 5.9	97.3 ± 6.5	0.60
Postoperative	22.9 ± 3.5	21.1 ± 2.4	0.08
Immediate postoperative correction rate (%)	76.5 ± 6.8	78.0 ± 9.2	0.58
Most recent follow-up	25.1 ± 3.7	24.6 ± 4.1	0.70
Correction rate at most recent follow-up (%)	74.1 ± 8.8	75.1 ± 10.5	0.89
Loss of correction	2.5 ± 1.2	2.8 ± 1.6	0.52
Thoracic kyphosis (°)			
Preoperative	17.2 ± 10.2	20.1 ± 12.2	0.83
Postoperative	24.9 ± 5.3	25.4 ± 6.2	0.44
Most recent follow-up	23.2 ± 4.6	24.6 ± 7.0	0.51
Coronal imbalance (mm)			
Preoperative	18.1 ± 8.5	19.4 ± 7.3	0.32
Postoperative	17.1 ± 7.3	18.4 ± 8.1	0.33
Final follow-up	15.3 ± 7.5	16.9 ± 8.2	0.20
Sagittal imbalance (mm)			
Preoperative	18.0 ± 10.2	18.3 ± 11.2	0.81
Postoperative	17.1 ± 8.3	17.4 ± 8.7	0.85
Final follow-up	13.3 ± 6.2	15.1 ± 9.4	0.63
Shoulder imbalance (mm)			
Preoperative	42.7 ± 10.9	45.1 ± 12.3	0.54
Postoperative	21.6 ± 6.8	23.4 ± 7.5	0.45
Final follow-up	18.4 ± 6.1	19.2 ± 7.2	0.72

*MSDR* multiple screws distraction reducer system, *ARPIDF* anterior release, posterior internal distraction, and subsequent spinal fusion

In the MSDR group, preoperative thoracic kyphosis of 17.2° ± 10.2° was corrected to 24.9° ± 5.3° at the immediate postoperative curve assessment and to 23.2° ± 4.6° at the most recent follow-up (Table 2). In the ARPIDF group, preoperative thoracic kyphosis of 20.1° ± 12.2° was corrected to 25.4° ± 6.2° at the immediate postoperative curve assessment and to 24.6° ± 7.0° at the most recent follow-up. There was no significant difference between the two groups with respect to preoperative, postoperative, and follow-up thoracic kyphosis.

In the MSDR group, preoperative and immediate postoperative coronal imbalance were 18.1 ± 8.5 and 17.1 ± 7.3 mm, respectively. At the most recent follow-up, it improved to 15.3 ± 7.5 mm. Preoperative and immediate postoperative sagittal imbalance were 18.0 ± 10.2 and 17.1 ± 8.3 mm, respectively. At the most recent follow-up, it improved to 13.3 ± 6.2 mm. Preoperative and immediate postoperative shoulder imbalance were 42.7 ± 10.9 and 21.6 ± 6.8 mm, respectively. At the most recent follow-up, it improved to 18.4 ± 6.1 mm. In the ARPIDF group, preoperative and immediate postoperative coronal imbalance were 19.4 ± 7.3 and 18.4 ± 8.1 mm, respectively. At the most recent follow-up, it improved to 16.9 ± 8.2 mm. Preoperative and immediate postoperative sagittal imbalance were 18.3 ± 11.2 and 17.4 ± 8.7 mm, respectively. At the most recent follow-up, it improved to 15.1 ± 9.4 mm. Preoperative and immediate postoperative shoulder imbalance were 45.1 ± 12.3 and 23.4 ± 7.5 mm, respectively. At the most recent follow-up, it improved to 19.2 ± 7.2 mm. There was no significant difference between the two groups with respect to preoperative, postoperative, and most recent follow-up coronal and sagittal balance.

In the ARPIDF group, 1 patient developed infection and 2 exhibited intraoperative neuromonitoring changes during correction maneuver; none of these events occurred in the MSDR group (*p* = 0.03). There were no transient neurologic complications or permanent neurologic deficit in either group. The preoperative and postoperative SRS-30 scores in the MSDR and ARPIDF groups are shown in Table 3. There is no significant difference in all preoperative domains between the MSDR and ARPIDF groups. The postoperative function and satisfaction scores in the MSDR group were significantly greater than those in the ARPIDF group, the between-group difference in other domains was not statistically significant. Transient dyspnea occurred in 1 patient after the initial surgery and resolved subsequently. No ventilator support was needed.

**Table 3:** Preoperative and postoperative SRS-30 scores of the two groups

	MSDR	ARPIDF	*p*-value
Pain			
Preoperative	4.1 ± 0.7	3.9 ± 1.0	0.49
Postoperative	4.3 ± 0.9	4.0 ± 0.8	0.30
Self-image			
Preoperative	3.2 ± 0.6	3.4 ± 0.7	0.36
Postoperative	4.0 ± 0.8	4.2 ± 0.6	0.62
Function			
Preoperative	3.3 ± 0.9	3.2 ± 0.8	0.72
Postoperative	4.4 ± 0.5	3.6 ± 0.6	0.03
Mental health			
Preoperative	3.6 ± 0.7	3.5 ± 0.5	0.63
Postoperative	4.1 ± 0.5	4.2 ± 0.8	0.65
Satisfaction			
Preoperative	3.8 ± 0.4	3.7 ± 0.3	0.40
Postoperative	4.3 ± 0.6	3.9 ± 0.5	0.03
Total score			
Preoperative	3.6 ± 0.7	3.5 ± 0.9	0.71
Postoperative	4.2 ± 0.8	4.0 ± 0.7	0.43

Data are presented as mean ± standard deviation

*MSDR* multiple screws distraction reducer system, *ARPIDF* anterior release, posterior internal distraction, and subsequent spinal fusion; SRS-30, 30-Item Scoliosis Research Society Questionnaire

## Discussion

Various correction methods have been developed to treat severe scoliosis [18]. Anterior release followed by posterior instrumented fusion has been widely reported in the literature [19]. The reported correction rates achieved with this method are 40–50%. PVCR enables translation and manipulation of both anterior and posterior spinal columns. However, PVCR necessitates aggressive osteotomies, which incur a greater risk of neurologic injury and blood loss. To achieve safer and easier correction of severe scoliosis, we previously performed staged anterior release, internal distraction, and posterior spinal fusion [15–17]. Our results demonstrated that scoliosis and rotation of the major curve are greatly corrected after the initial distraction. However, surgical procedures were performed in 2–3 stages, which increase operation and hospitalization times. In addition, anterior release may compromise pulmonary function and cause respiratory problems.

We designed a novel MSDR system to overcome the disadvantages of the current strategies used for the correction of severe spinal curve. The system takes advantage of a powerful manipulating arm that connects the proximal and distal spinal segments. Multiple rounds of distraction can be performed to achieve coronal correction and to translate the periapical vertebrae to the midline. Internal distraction has a similar effect as ARPIDF, but it involves a single-stage surgery. Following coronal correction, the hexagonal connector connected with the middle provisional rod is rotated through the threaded shaft to restore thoracic kyphosis for sagittal correction. The manipulation of the middle provisional rod helps achieve true axial derotation of the whole spine and chest wall. Subsequently, while MSDR is locked to hold the correction, apical vertebral translation and derotation are employed via the convex permanent rod. Because the periapical vertebrae are uncoupled from the rest of the spine, these are essentially unlocked and have greater freedom of movement; this provides an opportunity for the convex rod to effectively and safely induce sagittal translation and axial derotation at the apex.

**Fig. 3:**
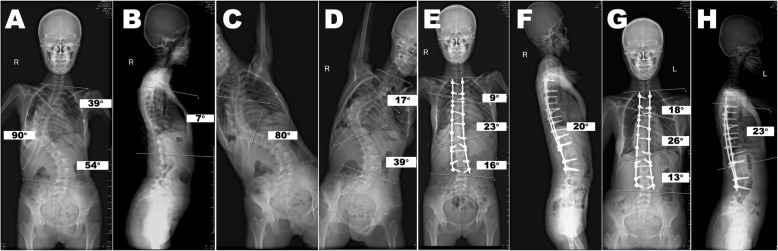
A girl aged 15 years and 3 months with a Lenke 4B curve. **a** The main thoracic curve T4–L1 Cobb angle was 90°. Further, C7PL–CSVL was 10 mm toward the right, and clavicle angle was 4°. The triradiate cartilage was closed, and the Risser stage was 4. **b** Sagittal T5–T12 kyphosis was 7°, lumbar lordosis T12–S1 was 52°, and C7PL–S1 was 5 mm. **c**, **d** The angle with standing side bending of the main thoracic curve was 80°, and flexibility was 11.1%. The patient underwent posterior spinal fusion from T2 to L3 with Ponte osteotomy using the rod-link-distraction-reducer (MSDR) system. **e** After MSDR correction and posterior spinal fusion, the major curve was corrected to 23°. The correction rate was 74.4%, and C7PL–CSVL was − 4 mm. **f** Further, sagittal T5–T12 kyphosis was 20°, lumbar lordosis T12–S1 was 53°, and C7PL–S1 was 3 mm. **g**, **h** At postoperative 2 years, the major curve was corrected to 26°; the correction rate was 71.1%, sagittal T5–T12 kyphosis was 23°, lumbar lordosis T12–S1 was 53°, and C7PL–S1 was − 3 mm. C7PL–CSVL, distance between the plumb line from the centroid of C7 (C7PL) and the central sacral vertical line (CSVL)

**Fig. 4:**
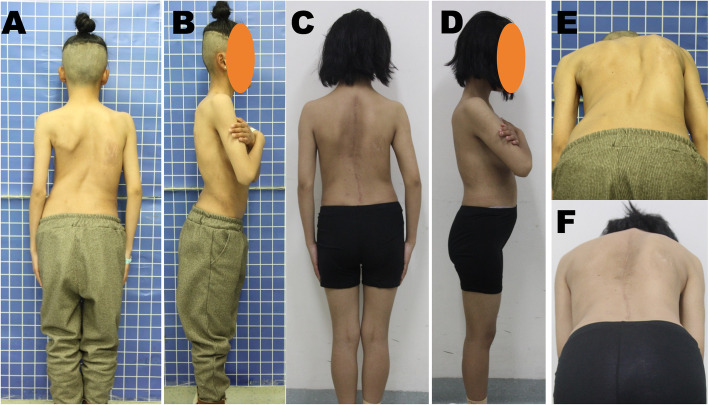
Panels **a**, **b**, and **e** show the preoperative clinical pictures and **c**, **d**, and **f** show the clinical pictures at postoperative 2 years

In our study (Figs. 3 and 4), the use of MSDR achieved similar curve correction as well as coronal and sagittal balance control compared to that of ARPIDF. The use of MSDR also significantly decreased operation and hospitalization times as well as reduced intraoperative blood loss. It facilitates easier surgery, which decreases the learning curve for the surgical treatment of severe scoliosis. Furthermore, the SRS-30 scores for function and satisfaction demonstrated greater improvement in the MSDR group than in the ARPIDF group. In addition, none of the patients in the MSDR group developed any respiratory complication or infection; however, there was 1 patient with dyspnea and 1 with wound infection in the ARPIDF group. This was likely related to open thoracic performance and multiple surgeries in ARPIDF.

Previously, a similar correction system was reported by Zhang et al. [20]. This system provides safer, easier, and better correction of severe curves without increasing the surgical risk. Our device was inspired by their design: reduction and correction were achieved using the manipulating arm connected to the proximal and distal provisional rods. However, in our system, provisional rods were placed on the concave side of the spine. This provided the initial internal distraction force for coronal correction and for translating the periapical vertebrae to the midline. Furthermore, we placed a middle provisional rod on the apical segment to address axial and sagittal deformities. This step ensures a true axial derotation of the whole spine and restores thoracic kyphosis for sagittal correction. In addition, compression and derotation of the convex permanent rod further decreases coronal and axial deformities. Similar to their results, the technical innovation helped achieve better clinical outcomes.

Certainly, the results of our study need to be interpreted with regard to its limitations. First, the retrospective nature of the study includes all inherent biases associated with this methodology. Second, this was a single-center study with a small sample size. Third, ARPIDF has been used previously at our institution. Lastly, the learning curve for surgical treatment of severe scoliosis may have had affected the results.

## Conclusions

The use of MSDR exhibited similar Cobb correction as that of ARPIDF. However, the former was associated with shorter operation and hospitalization times, lower blood loss, and lower complication rate. MSDR provides safer and easier correction of severe curves without increasing surgical risk.

## Data Availability

The datasets generated and/or analysed during the current study are not publicly available due to the patients’ privacy policy of West China Hospital but are available from the corresponding author on reasonable request.
